# Coordinate post-transcriptional repression of Dpp-dependent transcription factors attenuates signal range during development

**DOI:** 10.1242/dev.123273

**Published:** 2015-10-01

**Authors:** Fay G. Newton, Robin E. Harris, Catherine Sutcliffe, Hilary L. Ashe

**Affiliations:** Faculty of Life Sciences, University of Manchester, Manchester M13 9PT, UK

**Keywords:** BMP, Brat, Post-transcriptional repression, Pum, Differentiation, Mad, Medea, Schnurri

## Abstract

Precise control of the range of signalling molecule action is crucial for correct cell fate patterning during development. For example, *Drosophila* ovarian germline stem cells (GSCs) are maintained by exquisitely short-range BMP signalling from the niche. In the absence of BMP signalling, one GSC daughter differentiates into a cystoblast (CB) and this fate is stabilised by Brain tumour (Brat) and Pumilio (Pum)-mediated post-transcriptional repression of mRNAs, including that encoding the Dpp transducer, Mad. However, the identity of other repressed mRNAs and the mechanism of post-transcriptional repression are currently unknown. Here, we identify the *Medea* and *schnurri* mRNAs, which encode transcriptional regulators required for activation and/or repression of Dpp target genes, as additional Pum-Brat targets, suggesting that tripartite repression of the transducers is deployed to desensitise the CB to Dpp. In addition, we show that repression by Pum-Brat requires recruitment of the CCR4 and Pop2 deadenylases, with knockdown of deadenylases *in vivo* giving rise to ectopic GSCs. Consistent with this, Pum-Brat repression leads to poly(A) tail shortening and mRNA degradation in tissue culture cells, and we detect a reduced number of *Mad* and *shn* transcripts in the CB relative to the GSC based on single molecule mRNA quantitation. Finally, we show generality of the mechanism by demonstrating that Brat also attenuates pMad and Dpp signalling range in the early embryo. Together our data serve as a platform for understanding how post-transcriptional repression restricts interpretation of BMPs and other cell signals in order to allow robust cell fate patterning during development.

## Abstract (precis)

**Summary:** The translational repressors Brat and Pumilio attenuate Dpp signalling range in the *Drosophila* female germline and early embryo to ensure precise cell fate patterning.

## INTRODUCTION

The bone morphogenetic proteins (BMPs) constitute a major conserved family of signalling proteins that regulate a range of cellular processes during development and homeostasis, including cell fate determination (Ramel and Hill, 2012). For example, in *Drosophila* the BMP signalling molecule Dpp patterns fates over a range of cell diameters in different developmental contexts (Affolter and Basler, 2007), with ovarian germline stem cells (GSCs) maintained in response to an exquisitely short-range Dpp signal. Each *Drosophila* ovary consists of 16-20 ovarioles and at the tip of each ovariole is the germarium (Fig. 2A), a structure containing two or three GSCs that reside within a surrounding somatic cell niche (Harris and Ashe, 2011; Eliazer and Buszczak, 2011). Dpp released from the niche is highly restricted in range through sequestration by extracellular collagen IV (Wang et al., 2008) and the requirement for the niche-associated glypican Dally for Dpp stability (Guo and Wang, 2009; Liu et al., 2010). The extracellular Dpp signal is transduced within GSCs through the activation of the Mothers against dpp (Mad) and Medea (Med) transcription factors. Phosphorylated Mad (pMad) forms a complex with Med that activates Dpp target genes, whereas recruitment of the Schnurri (Shn) corepressor to the pMad-Med complex confers transcriptional repression (Hamaratoglu et al., 2014). In GSCs, the pMad-Med-Shn complex directly represses transcription of *bag of marbles* (*bam*), which encodes a key differentiation factor (Chen and McKearin, 2003a,b; Pyrowolakis et al., 2004), as well as *fused*, encoding a kinase that phosphorylates the Dpp receptor, targeting it for proteasomal degradation (Xia et al., 2010, 2012). When a GSC divides, one daughter remains within the niche while the other, the cystoblast (CB) is displaced posteriorly and consequently receives a lower level of Dpp signal. This results in de-repression of Bam and subsequent differentiation (Kirilly and Xie, 2007; Harris and Ashe, 2011).

Translational controls also play important roles in regulating the balance between GSC self-renewal and differentiation. Like other stem cells, *Drosophila* GSCs are maintained by two major translational repressors: the Puf proteins Pumilio (Pum) and Nanos (Nos) (Gilboa and Lehmann, 2004; Wang and Lin, 2004). One target of Pum-Nos repression within GSCs is *brain tumor* (*brat*) mRNA, encoding a translational repressor that acts as a differentiation factor in both the female germ line and in neural stem cells (Betschinger et al., 2006; Lee et al., 2006; Harris et al., 2011). Recently it has been shown that Pum-Nos also repress *mei-P26* mRNA in GSCs by recruiting the CCR4-NOT deadenylation complex (Joly et al., 2013). Mei-P26 is required at low levels in GSCs and plays a role in self-renewal through repressing translation of Brat as part of a Pum-Nos-Mei-P26 complex (Li et al., 2012). In contrast, Mei-P26 is expressed at high levels in CBs and differentiating cysts where it promotes differentiation and restricts proliferation through inhibition of the miRNA pathway (Neumüller et al., 2008).

Translational repression also plays a significant role in maintaining CB fate and promoting differentiation. Together with the RNA helicase Bgcn, and potentially also Sex-lethal and Mei-P26 (Chau et al., 2009; Li et al., 2013), Bam represses translation of Nos in the CB (Li et al., 2009) thereby relieving repression of differentiation factors such as Brat. Brat itself is able to form a complex with Pum (Sonoda and Wharton, 2001) and post-transcriptionally repress mRNAs encoding both Mad and the growth factor Myc in CBs (Harris et al., 2011). As CBs are born relatively close to the niche, repression of *Mad* mRNA might be important to reduce Mad protein levels in CBs, thereby ensuring that *bam* is transcribed even in the presence of low levels of Dpp signal. In addition to Pum-Brat repression, other mechanisms restrict Dpp signal transduction in CBs, including Fused and Smurf-dependent targeting of the Dpp receptor Thickveins to the proteasome (Xia et al., 2010, 2012) and depletion of the mRNA encoding the BMP receptor Saxophone by miRNA repression (Iovino et al., 2009). However, despite the importance of Pum-Brat repression in this system, relatively little is known about the precise mechanism of repression.

In this work we present evidence that, in addition to Mad, Pum-Brat also post-transcriptionally repress other BMP signal transducers. In addition, we provide mechanistic detail underpinning Pum-Brat repression of target mRNAs by showing a requirement for deadenylases. Furthermore, we show that Brat represses Mad expression in the embryo, demonstrating that antagonism between Brat repression and BMP signalling also exists in other developmental contexts.

## RESULTS

### Pum and Brat post-transcriptionally regulate the *Med* and *shn* mRNAs

**Fig. 1.**
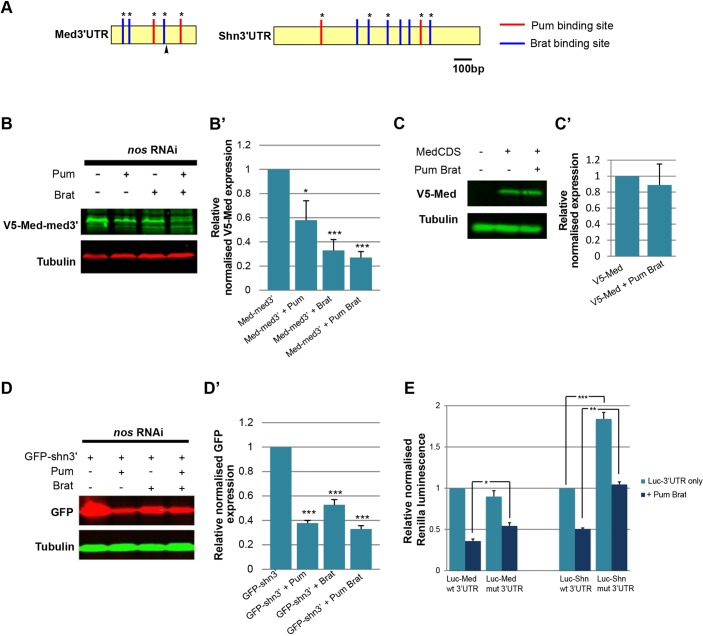
**Pum-Brat post-transcriptional repression via the *Med* and *shn* 3′** **UTRs.** (A) Schematic of the *Med* and *shn* 3′ UTRs showing the position of sequences matching the Pum binding site consensus [U(G/U/A)(U/A)AN(A/C/U)(A/G)] and the Brat binding site (GUUGU, UGUUA or UGUUU). Asterisks denote mutated sites. The arrowhead denotes an alternative shorter 3′ UTR for Med. (B) Representative western blot showing Pum-Brat repression of V5-tagged Med-med3′ in transfected S2 cells, with (B′) quantification of GFP expression in three biological repeats normalised relative to tubulin. Error bars show s.d., *P<0.05, ***P<0.001. (C) Representative anti-V5 western blot showing no repression of V5-Med is observed in the absence of *Med* 3′ UTR, with (C′) quantitation as in B′. (D) Representative western blot showing Pum-Brat repression of GFP-shn3′ in transfected S2 cells, with quantitation as in B′ of three biological repeats (D′). Error bars show s.d., ***P<0.001. (E) Graph showing *Renilla luciferase* expression, relative to *Firefly luciferase*, from cells transfected with plasmids carrying the wild type and mutated *Med* and *shn* 3′ UTRs downstream of *Renilla*. The sites mutated correspond to those labelled with an asterisk in A. Experiments were performed in the presence and absence of Pum-Brat, as labelled, *n*=3. Error bars show s.d.; *P<0.05, **P<0.01, ***P<0.001.

Dpp-dependent transcriptional activation is mediated by a complex of transcription factors pMad and Med, whereas a pMad-Med-Shn complex mediates transcriptional repression (Hamaratoglu et al., 2014). As we have previously provided evidence that Pum-Brat post-transcriptionally represses *Mad* mRNA via its 3′ UTR (Harris et al., 2011) we wished to determine whether similar regulation also exists for *Med* and *shn* mRNAs. Initially, we analysed the *Med* and *shn* 3′ UTR sequences for matches to the Pum binding consensus (Gerber et al., 2006) and the recently identified Brat binding site (Loedige et al., 2014; Laver et al., 2015). This revealed multiple matches to both in each 3′ UTR (Fig. 1A), suggesting that these might be potential regulatory targets.

We next tested whether these 3′ UTRs confer post-transcriptional regulation in a tissue culture assay. A tagged construct bearing the *Med* coding sequence and both the 5′ and 3′ UTRs (Med-med3′) was transfected into *Drosophila* S2 cells, in the absence or presence of Pum and/or Brat. S2 cells express Pum, Nos and Brat (modENCODE RNAseq data, Graveley et al., 2011). However, as *Mad* mRNA regulation by Pum-Brat occurs in the absence of Nos in the CB, we mimicked this situation by reducing Nos levels in S2 cells using *nos* RNAi, as described previously (Harris et al., 2011). Co-transfection of Pum and Brat significantly reduced expression compared to Med-med3′ alone (Fig. 1B,B′) and no repression was observed in the absence of the *Med* 3′ UTR (Fig. 1C,C′). This suggests that *Med* mRNA might be post-transcriptionally regulated by Pum and Brat, in a manner similar to that we have described for *Mad* mRNA (Harris et al., 2011). We next tested whether *shn* expression is also repressed post-transcriptionally by expressing a GFP construct bearing the *shn* 3′ UTR (GFP-shn3′) in S2 cells in the presence of *nos* RNAi. GFP-shn3′ expression was repressed when co-transfected with either Pum or Brat (Fig. 1D,D′), in contrast to the GFP control that showed no repression by Pum and Brat (data not shown; Harris et al., 2011).

To determine the contribution to this regulation of the identified Pum or Brat sites in the *Med* and *shn* 3′ UTRs (Fig. 1A), we carried out site directed mutagenesis of two Pum and three Brat sites in each 3′ UTR. In the case of the *shn* 3′ UTR, the three Brat sites chosen included one matching the consensus described by Loedige et al. (2014) (GUUGU) and two matching the consensus identified by Laver et al. (2015) by both RIP-chip and RNAcompete (UGUUA), whereas the three remaining sites match the consensus identified by RIP-chip only. Wild-type and mutated UTRs were cloned into a dual luciferase reporter vector and transfected into S2 cells. With the *Med* 3′ UTR, the relative Renilla expression decreases upon Pum and Brat addition (Fig. 1E), mirroring the results obtained with the Med-med3′ reporter (Fig. 1B). The degree of repression is significantly reduced when the Pum and Brat sites are mutated (Fig. 1E), but not abolished, which might reflect incomplete disruption of binding when Pum and Brat are overexpressed, the presence of additional cryptic sites or that the 3′ UTR can be regulated through another mechanism. When testing the regulation of the *shn* 3′ UTR, we found that addition of Pum-Brat to the wild-type 3′ UTR repressed expression as expected. However, compared to the wild type, the mutant 3′ UTR was significantly de-repressed in both the absence and presence of Pum-Brat, comparable to the ∼2 fold effect recently reported in a similarly designed repression assay in which Brat binding sites were also mutated (Laver et al., 2015). Taking into account this baseline de-repression, we detected no difference in Pum-Brat repression of the mutant 3′ UTR versus the wild type, which we speculate is because of the presence of additional sites that we have not mutated (Fig. 1A). Unlike the wild-type *shn* 3′ UTR, we do not observe de-repression of the wild-type *Med* 3′ UTR in the absence of added Pum and Brat when the Pum-Brat sites are mutated, which we attribute to the endogenous levels of Brat being insufficient to repress the *Med* 3′ UTR. Consistent with this, the effect on the Med-med3′ reporter when Pum alone is added is weaker and less significant than that observed when only Pum is added to the GFP-shn3′ reporter (compare Fig. 1B′ and D′).

### Brat repression of Med and Shn *in vivo*

**Fig. 2.**
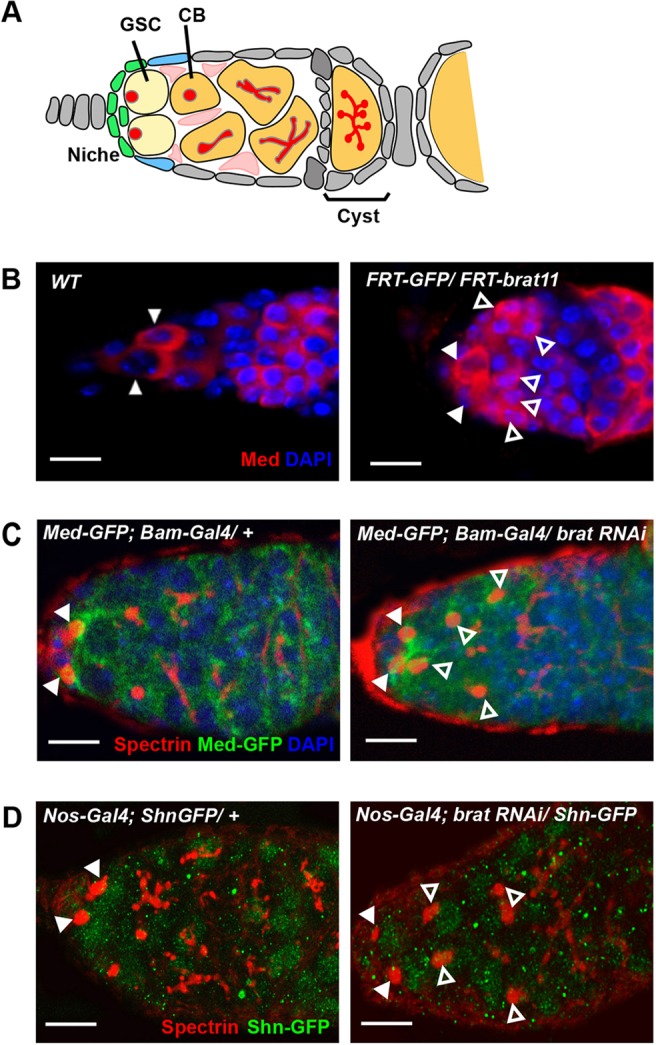
**Ectopic expression of Med and Shn in *brat* mutant germaria.** (A) Structure of the *Drosophila* germarium: GSC, germline stem cell; CB, cystoblast; spectrosome (round) and fusome (branched) organelles are depicted in red. (B) Med expression is restricted to GSCs in wild type germaria (closed arrowheads) and expanded in germaria containing *brat^11^* mutant germline clones (open arrowheads). Scale bars: 6 μm. (C) Med-GFP (green) expression in a wild type germarium is high in GSCs and typically localized around the spectrosome (red, visualised with anti-Spectrin). The ectopic GSC-like cells observed upon knockdown of *brat* in CBs with *bam-Gal4VP16* driving the *brat* shmiR are also Med-GFP positive. Closed arrowheads indicate GSCs, ectopic GSC-like cells are labelled with open arrowheads. Scale bars: 10 μm. (D) Anti-GFP (to detect Shn-GFP, green) and anti-Spectrin (red) staining in wild-type and *brat* knockdown germaria. Shn is expressed in GSCs (closed arrowheads) but not CBs in wild type, whereas in *brat* knockdown germaria additional cells with round spectrosomes are also positive for Shn-GFP (open arrowheads). Scale bars: 10 μm.

To test whether post-transcriptional repression of Med and Shn by Brat is relevant *in vivo*, we visualised Med protein in germaria from wild-type flies and those with *brat* mutant germ lines (generated by tissue-specific flipase mediated mitotic recombination). In wild-type germaria Med is present at higher levels in GSCs than CBs (Fig. 2B), consistent with a requirement for active Dpp signalling in these cells. In contrast, there is an expanded pattern of Med expression in a germline mutant for the *brat^11^* null allele (Fig. 2B) and Med-GFP staining when Brat expression was knocked down in the CB using *bam*-*Gal4VP16* to drive a *brat* shRNA (Fig. 2C), consistent with Brat acting as a repressor of Med expression in the ovary. We also visualised Shn protein in the germarium using a Shn-GFP fusion, which is present at higher levels in GSCs than CBs in wild-type ovaries (Fig. 2D). However, when Brat expression was knocked down using a *nos-Gal4VP16* driven *brat* shRNA transgene, we observed expanded Shn-GFP expression in cells bearing round spectrosome organelles (Fig. 2D), providing *in vivo* support for *shn* mRNA also being post-transcriptionally repressed by Brat.

### A conserved tryptophan residue within Pum is important for repression of *Mad* mRNA 3′ UTR

**Fig. 3.**
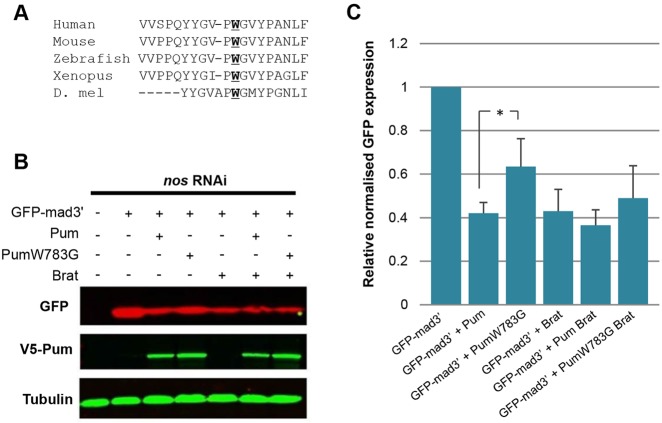
**A conserved Trp residue in Pum is important for Pum-Brat repression.** (A) Sequence alignment of a conserved region in *Drosophila* Pum and human, mouse, zebrafish and *Xenopus* Pum2 proteins, showing a conserved Trp residue (W783 in *D. melanogaster*) required for the repressive function of Pum. (B) Representative western blot showing reduced repression of GFP-mad3′ by PumW783G in S2 cells. V5-tagged Pum and PumW783G are expressed at approximately equal levels in these samples. (C) Shows the quantitation of four biological repeats, normalised relative to tubulin. Error bars represent the s.d., the W783G mutation results in significant loss of repression (*P<0.05 by *t*-test) compared with wild-type Pum.

To determine the mechanism of Pum-Brat post-transcriptional repression, we focused on regulation of *Mad* mRNA. In terms of a potential effect of Pum on translation initiation, studies in *Xenopus* have shown that Pum2 represses translation by competing with eIF4E for binding to the 5′ 7-methyl guanosine cap (5′7 mG), thereby blocking translation initiation. In addition, the ability of Pum2 to out-compete eIF4E requires a conserved Trp residue (W344 in *Xenopus*) (Cao et al., 2010). As this Trp residue is also conserved in *Drosophila* (W783, Fig. 3A) we investigated whether mutation of W783 to Gly affected the ability of Pum to repress translation.

A GFP-reporter construct bearing the *Mad* 3′ UTR (GFP-mad3′), which we have used previously as a reporter of Pum-Brat repression (Harris et al., 2011), was transfected into *Drosophila* S2 cells along with Pum, Brat or mutant Pum (PumW783G) expression plasmids, in the presence of *nos* RNAi. Western blot analysis revealed repression of GFP-mad3′ by Pum. However, we observed a significant reduction in this repression when the PumW783G construct was transfected (Fig. 3B,C), even though the wild-type and mutant Pum proteins accumulate to similar levels (Fig. 3B), suggesting that this residue is important for full repression. Transfection of Brat alone resulted in a similar level of repression to Pum-Brat co-transfection, suggesting that there might be sufficient endogenous Pum in S2 cells to allow repression with the addition of Brat, masking the effect of the transfected Pum. Consistent with this, transfection of PumW783G together with Brat only relieves repression moderately, with the repression observed potentially as a result of the activity of endogenous wild-type Pum and Brat. However, together these data suggest that the W783 residue is required for Pum to fully repress translation, and that *Drosophila* Pum might act in a similar manner to vertebrate Pum2, blocking translation through preventing eIF4E from binding to the 5′ 7 mG cap.

### Repression by Pum-Brat requires recruitment of deadenylases

**Fig. 4.**
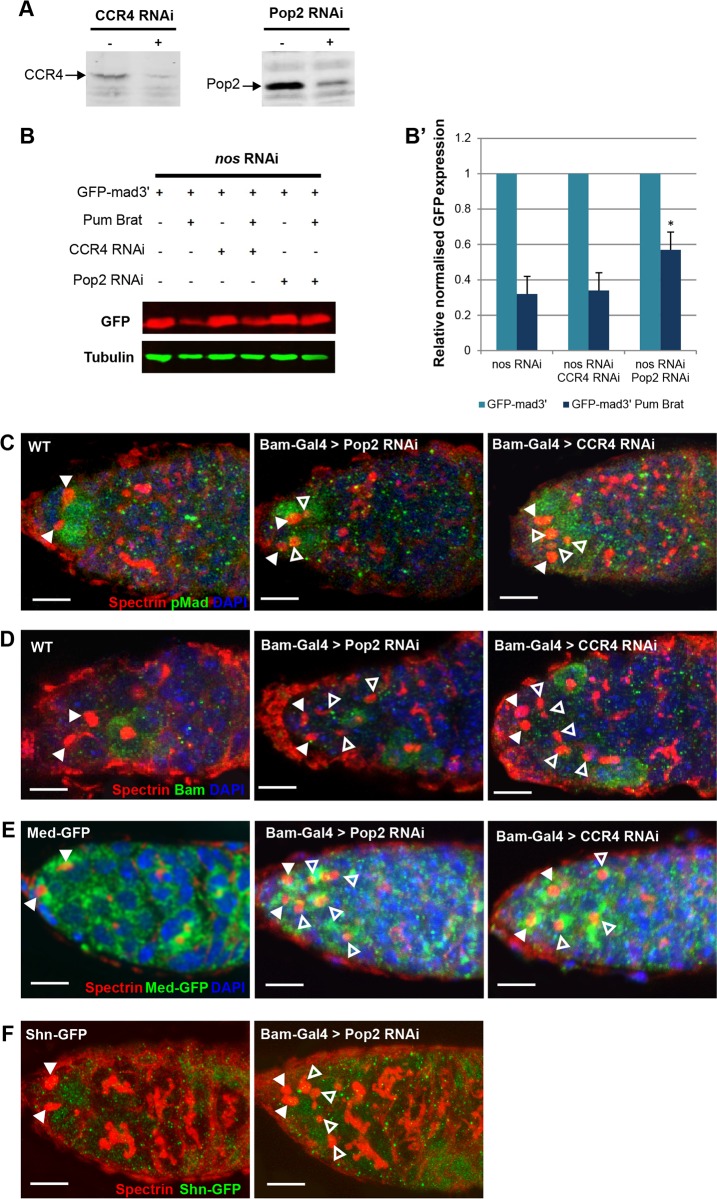
**The CCR4 and Pop2 deadenylases are required for timely differentiation of GSC daughters.** (A) Western blots showing that CCR4 and Pop2 protein expression is efficiently knocked down by dsRNAs. (B) Representative western blot showing the effect of RNAi knockdown of CCR4 and Pop2 deadenylases on Pum-Brat repression of GFP-mad3′ in S2 cells. Three biological repeats are quantified in (B′), there is a significant reduction in Pum-Brat repression when Pop2 is knocked down (*P<0.05 by *t*-test, error bars represent s.d.). (C,D) Wild-type and CCR4 or Pop2 deadenylase knockdown germaria stained with anti-Spectrin (red) and in green – anti-pMad (C) and anti-Bam (D). CCR4 and Pop2 shRNA expression was driven specifically in CBs by *bam-Gal4VP16*. (E,F) As in C except that the germaria carry Med-GFP (E) and Shn-GFP (F), with anti-GFP staining in green. Closed arrowheads indicate GSCs, open arrowheads indicate additional GSC-like cells with round spectrosomes. Scale bar: 10 μm.

In addition to effects on initiation, repression of protein levels can often correlate with the deadenylation of mRNAs (Wiederhold and Passmore, 2010). In the *Drosophila* embryo and GSCs, there is evidence that the inhibition of translation by Pum and Nos involves recruitment of the CCR4-NOT deadenylase complex (Kadyrova et al., 2007; Joly et al., 2013). Therefore, we investigated whether the CCR4-NOT complex deadenylases play a role in Pum-Brat post-transcriptional repression. In *Drosophila* the CCR4-NOT complex is composed of seven proteins including NOT1-NOT4 and two deadenylases Pop2 (also known as CAF1) and CCR4 (Barckmann and Simonelig, 2013; Temme et al., 2010). Initially we used RNAi to knock down CCR4 and Pop2 expression in S2 cells, followed by transfection of GFP-mad3′ with and without Pum and Brat. Successful reduction in CCR4 and Pop2 protein levels by RNAi was verified by western blot (Fig. 4A). Visualisation of GFP levels revealed that knockdown of Pop2 caused significant loss of Pum-Brat repression of GFP-mad3′, whereas knockdown of CCR4 had no effect (Fig. 4B,B′). Although Joly et al. (2013) observed that CCR4 is the crucial deadenylase interacting with Pum in GSCs, our results here are consistent with Pop2 playing a more dominant role in the CCR4-NOT complex in S2 cells (Temme et al., 2010). It is interesting that only partial loss of repression was observed, however this might be a result of incomplete knockdown of Pop2 (Fig. 4A). One explanation for the requirement for Pop2 is that Pum-Brat could recruit the deadenylase complex to the target mRNA to promote its degradation through removal of the poly(A) tail.

To further investigate the role of the deadenylases, we tested whether there was a requirement for either CCR4 or Pop2 for CB differentiation *in vivo*. shRNA transgenes for either *CCR4* (also known as *twin*) or *Pop2* were used to knock down expression of these deadenylases in CBs by driving shRNA expression with *bam-Gal4VP16*. Knockdown of either deadenylase resulted in an increased number of cells with round spectrosome organelles in the germarium (Fig. 4C-F). Wild type germaria have on average four cells bearing round spectrosomes in each germarium, whereas we observed an average of 6.0±0.93 cells per germarium when CCR4 was knocked down (*n*=20) and 6.15±0.80 cells per germarium when Pop2 was knocked down (*n*=15). The additional cells bearing round spectrosomes in CCR4 and Pop2 knockdown germaria were pMad positive and Bam negative and therefore resemble undifferentiated GSC-like cells (Fig. 4C,D). As loss of either deadenylase in the complex is sufficient to cause the additional GSC-like cell phenotype it would appear that CCR4 and Pop2 do not function redundantly and it is possible that neither deadenylase is expressed at sufficiently high level to compensate for loss of the other.

The increase in pMad levels might reflect higher *Mad* mRNA levels, as would be predicted if Pum-Brat repression involves recruitment of the CCR4-NOT complex. Consistent with this, we also observed ectopic expression of both Med-GFP and Shn-GFP in the additional GSC-like cells when CCR4 and Pop2 were knocked down (Fig. 4E,F). The up-regulation of Mad, Med and Shn protein levels provides a molecular explanation for the ectopic GSC-like cells observed, as a result of continued repression of *bam* transcription outside of the niche. Taking these results together with our observations in S2 cells, we hypothesise that in the absence of deadenylase activity, *Mad*, *Med* and *shn* transcripts persist in germline cells outside of the niche, allowing the persistence of Dpp signalling that results in delayed onset of Bam expression, and subsequent differentiation. Deadenylation might therefore be required to destabilise Mad transcripts hastening their degradation and removal from CBs.

### Pum-Brat targeted transcripts undergo poly(A) shortening and degradation

**Fig. 5.**
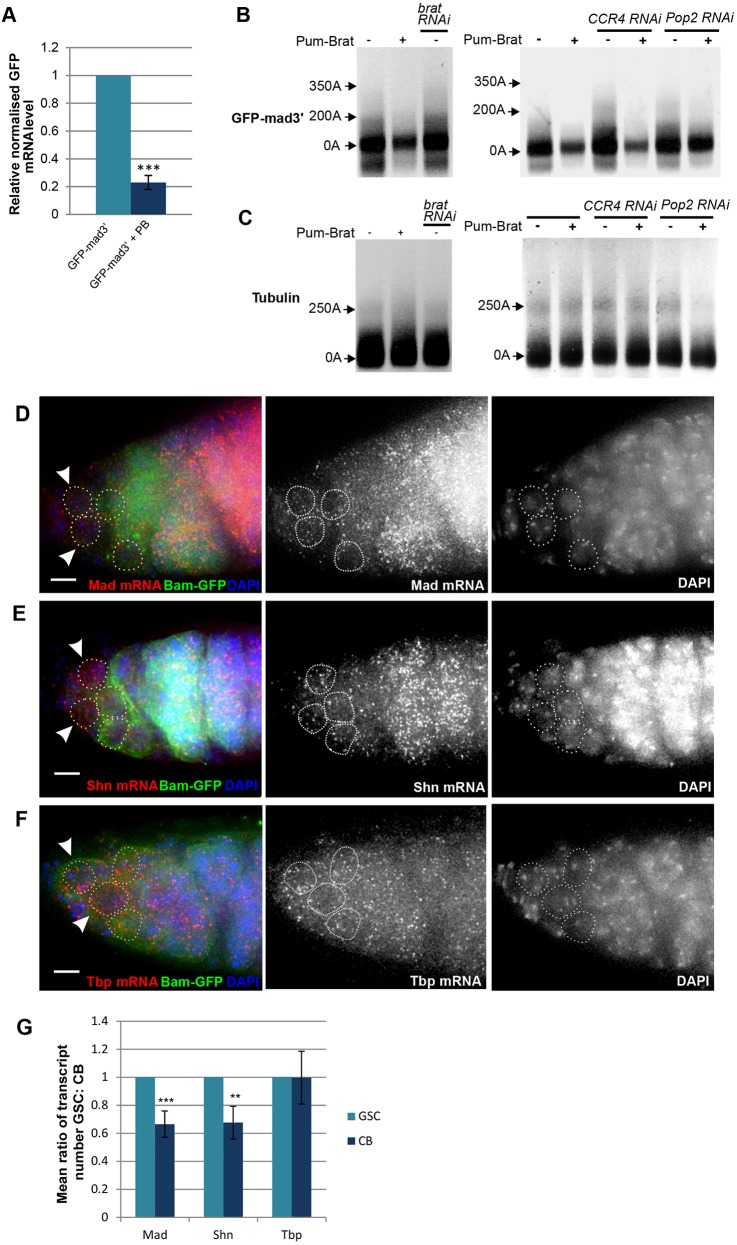
**Pum-Brat repression leads to deadenylation and degradation of target mRNAs.** (A) Quantification of GFP-mad3′ mRNAs in S2 cell extracts by qPCR. Graph shows the normalised mean of three biological repeats relative to Rp49, there is a 78% reduction in GFP-mad3′ mRNA level following Pum-Brat (PB) repression (error bar represents s.d., ***P<0.001 by *t*-test). (B) RACE-PAT assay shows poly(A) tail length of *GFP-mad3′* mRNAs following Pum-Brat repression and/or treatment with *brat*, *CCR4* or *Pop2* RNAi in S2 cells. (C) There is no reduction in poly(A) tail length of *beta-tubulin* mRNAs extracted from the same transfected S2 cells. (D-F) *Mad* (D), *shn* (E) and *Tbp* (F) mRNAs detected by Stellaris fluorescence *in situ* hybridisation (red) in representative *bam-GFP*; *vasa-GFP* germaria co-stained with anti-GFP (green). Dotted lines indicate GSCs (white arrows) and CBs (Bam-positive); scale bar: 5 μm. (G) Ratio (GSC:CB) of the mean number of *Mad*, *shn* and *Tbp* transcripts per cell in each germarium, normalised to the number in GSCs (*n*=13). Transcripts were quantified in GSCs and CBs using Imaris software. There is a significant reduction in *Mad* and *shn*, but not *Tbp*, transcript number in CBs versus GSCs (**P<0.01, ***P<0.001 by *t*-test, error bar represents s.d.).

Whereas some evidence exists for deadenylation-independent roles of the CCR4-NOT complex in translational repression (Chekulaeva et al., 2011; Cooke et al., 2010; Weidmann et al., 2014), more generally it has been proposed that this complex reduces translation and destabilises mRNA in a process reliant upon poly(A) tail removal (Temme et al., 2004; Chicoine et al., 2007). In order to assess whether repression by Pum-Brat leads to a loss of mRNA transcripts, we initially measured GFP-mad3′ transcript levels in S2 cells, with and without Pum-Brat co-transfection. The level of the GFP-mad3′ transcript in samples derived from cells co-transfected with Pum and Brat was reduced by 80% compared to controls (Fig. 5A). This decrease in mRNA levels implies that Pum-Brat mediated repression involves mRNA degradation. Furthermore, PAT-assays reveal that the poly(A) tail length of transcripts bearing the *Mad* 3′ UTR decreases in the presence of Pum-Brat (Fig. 5B). Although the majority of transcripts detected in both cases have a tail length of less than 50A because of preferential PCR amplification of shorter transcripts, the maximum tail length detected in unrepressed samples was 200A whereas in Pum-Brat repressed samples this reduces to around 70A. Poly(A) tail length also increased to around 350A when endogenous Brat was knocked down by RNAi prior to transfection (Fig. 5B). Knockdown of either CCR4 or Pop2 by RNAi resulted in a similar increase in poly(A) tail length and knockdown of Pop2 in particular resulted in a greater number of transcripts retaining longer tails in the presence of Pum and Brat (Fig. 5B). In contrast we found no difference in poly(A) tail length of tubulin transcripts in the same samples (Fig. 5C). These data suggest that Pum-Brat recruitment to the *Mad* 3′ UTR leads to a shortening of poly(A) tail length and transcript instability and this process requires the CCR4-NOT complex deadenylases.

To determine whether Mad transcripts are also degraded in CBs we used Stellaris fluorescence *in situ* hybridisation to label and quantify Mad transcripts within GSCs and CBs in wild type ovaries. GSCs were identified from their position within the niche and CBs identified as cells expressing Bam (Fig. 5D). GSCs had on average 50.8±13.8 Mad transcripts per cell whereas the mean number of transcripts in CBs was 33.0±8.0. The number of Mad transcripts detected in CBs ranged from 50% to about 80% of the number detected in GSCs in the same germarium meaning that, on average, there was a 35% reduction in the number of Mad transcripts between GSCs and CBs (*P*<0.01, *n*=13; Fig. 5D,G). Similarly, we found that the mean number of *shn* transcripts decreased from 42.3±7.2 in GSCs to 28.2±6.9 in CBs with, on average, a 32% reduction in the number of transcripts detected in CBs versus GSCs in the same germarium (*P*<0.01, *n*=12; Fig. 5E,G). It is possible that the variability observed relates to the age of the CBs when the samples are fixed. The transcript number would be expected to be similar in both daughters immediately after GSC division, with any effect of Brat on deadenylase recruitment and transcript destabilisation first requiring time for the Brat protein to accumulate in the CB. Despite the variability, we did observe a statistically significant decrease in both *Mad* and *shn* mRNA levels in CBs versus GSCs. In contrast, we found no significant difference in the number of TATA-binding protein (TBP) transcripts between GSCs and CBs despite *Tbp* being expressed at comparable levels to *Mad* and *shn* in the germarium (36.0±7.8 transcripts in GSCs and 34.7±8.5 transcripts in CBs, *n*=11; Fig. 5F,G). This suggests that the observed reduction in *Mad* and *shn* transcripts is specific to transcripts that are repressed in CBs. Therefore, together our data support a model whereby Pum-Brat recruit deadenylases to key target mRNAs, leading to poly(A) tail shortening and destabilisation.

### Brat restricts Mad expression and Dpp signalling in the early embryo

**Fig. 6.**
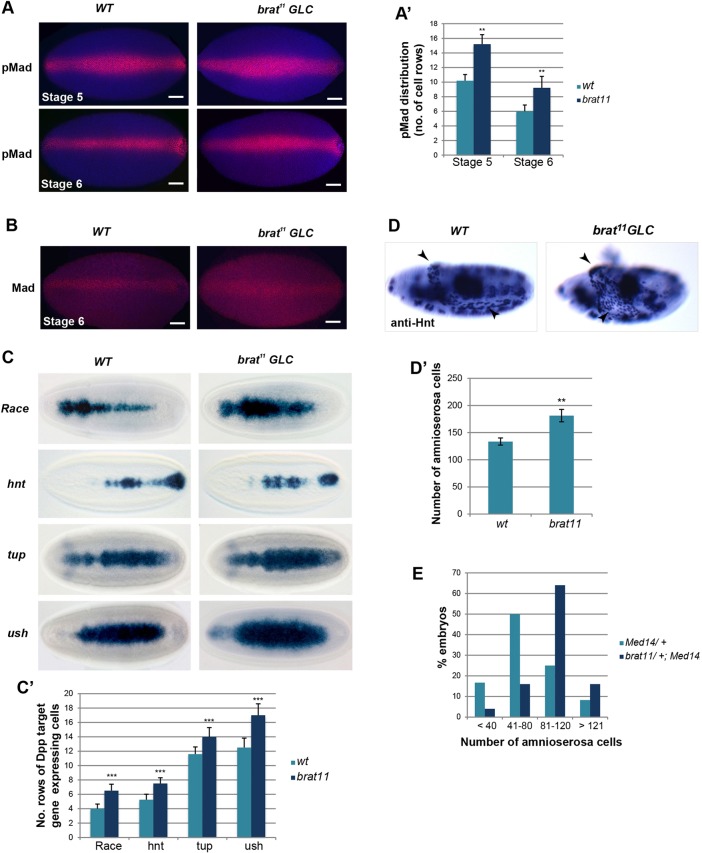
**Brat limits Dpp signalling in the embryo.** (A) pMad distribution in wild-type and *brat^11^* GLC embryos, with quantitation shown in (A′); *n*=9, error bars represent s.d., **P<0.01; (B) Mad protein levels visualised in wild-type and *brat^11^* GLC embryos. (A,B) Scale bars: 50 μm. (C) RNA *in situ* hybridisation of Dpp target genes in wild-type and *brat^11^* GLC embryos. Expression of *Race*, *hnt*, *tup* and *ush* is expanded in *brat* mutant embryos, quantitation is shown in C′. *n*=10, error bars represent s.d., ***P<0.001. (D) Representative wild-type and *brat^11^* GLC embryos stained with anti-Hnt to visualise amnioserosa cells (arrowheads), with quantitation in (D′). *n*=7, error bars represent s.d., **P<0.01. (E) Graph showing the proportion of embryos from either *Med*^*14*^/+ × *Med*^*14*^/*TM3* or *brat^11^/+; Med^14^/+*×*Med^14^/TM3* crosses that fall into different categories relating to the number of amnioserosa cells. Amnioserosa cells were visualised with anti-Hnt. The graph shows the proportion of embryos in each category (*n*=25).

The data presented thus far are consistent with Pum-Brat repressing *Mad*, *Med* and *shn* mRNAs in CBs to attenuate Dpp signal transduction. Given that these factors are co-expressed at other developmental timepoints, we were interested in whether this regulation might occur during other stages. As Pum and Brat are expressed in the early embryo (Sonoda and Wharton, 2001), where a Dpp gradient plays a key role in DV patterning (O'Connor et al., 2006), we tested whether Brat affects Dpp signalling in this context. To this end, *brat^11^* homozygous mutant germline clones were induced in females using the FLP-recombinase dominant female sterile technique (Chou and Perrimon, 1996; see Materials and Methods), to produce maternally null embryos (*brat^11^* GLC). Visualisation of activated pMad in these embryos by immunostaining reveals an expansion in *brat^11^* GLC embryos compared with wild type (Fig. 6A). This expansion, although statistically significant at stages 5 and 6 (Fig. 6A′), became less marked in older embryos (stage 6) in terms of number of cell widths, which might reflect the build up of Mad protein levels over time, counteracting the repression, or the decline in maternally expressed Pum protein levels at stage 6. A similar phenotype is observed when an antibody to Mad, rather than pMad, is used (Fig. 6B). These data suggest that in the absence of Brat, Mad protein levels are sufficiently high to allow activation in regions that receive lower levels of Dpp signal, resulting in a broader stripe of activated pMad.

To determine whether the expanded pMad peak affects Dpp-dependent expression patterns, we examined the expression patterns of several known Dpp target genes by *in situ* hybridisation. Expression widths of *Race* (also known as *Ance*), *hindsight* [*hnt*, also known as *pebbled* (*peb*)], *tailup* (*tup*) and *u-shaped* (*ush*) mRNAs showed a significant increase in *brat^11^* GLC embryos compared with wild type (Fig. 6C,C′). Peak levels of Dpp signalling specify amnioserosa tissue (Ferguson and Anderson, 1992), a fate typically adopted by 130 cells in wild-type embryos (Wharton et al., 1993; Miles et al., 2008). Consistent with the expanded expression of Dpp target genes in *brat^11^* GLC embryos, we also observed a significant increase in the number of amnioserosa cells from 133.6±6.5 in wild-type to 181.3±11.5 in *brat^11^* GLCs (Fig. 6D,D′). We also counted amnioserosa cell number to investigate a genetic interaction between the *brat^11^* and *Med^14^* loss-of-function alleles. To this end, we analysed progeny from females that were either *Med^14^*/+ or *brat^11^*/+; *Med^14^*/+ crossed to *Med^14^* heterozygous males. When the females were *Med^14^*/+, embryos showed variable reductions in amnioserosa number, consistent with a reduced Dpp signalling response. However, we found that the phenotype could be partially rescued when the females were also heterozygous for the *brat^11^* mutation, indicated by a greater proportion of embryos with a higher number of amnioserosa cells (Fig. 6E), consistent with antagonism between Brat and the Dpp pathway. Taken together, these results show that Brat has a negative regulatory effect on Dpp signalling in the early embryo, consistent with a general role for Brat as a post-transcriptional repressor of the Dpp signal transduction pathway in different developmental contexts.

## DISCUSSION

**Fig. 7.**
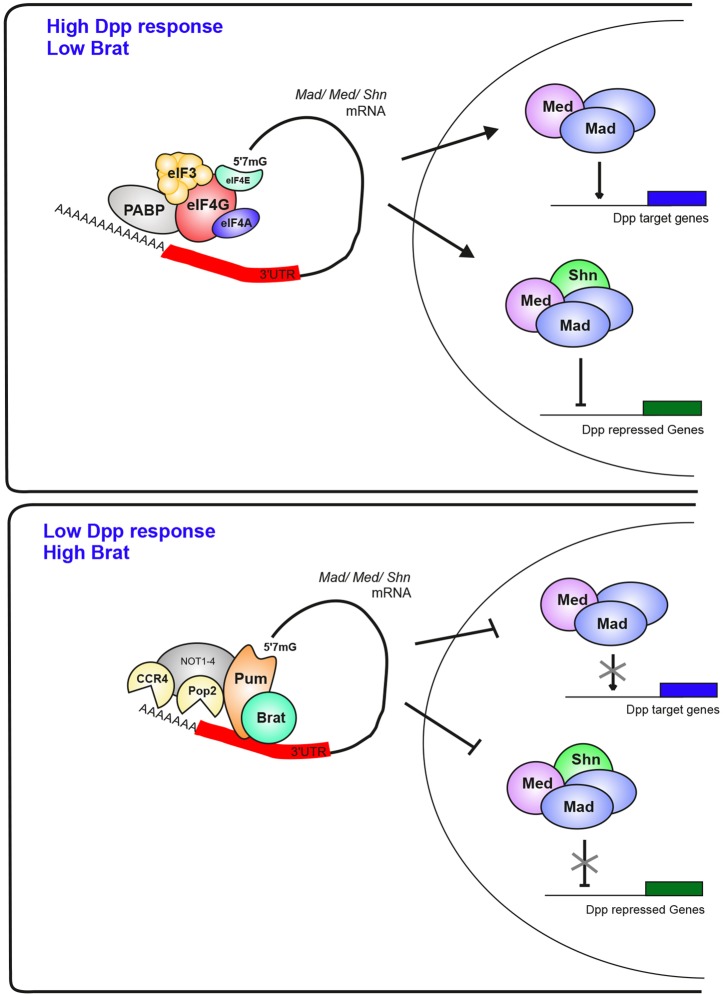
**Model of Pum-Brat repression in attenuating Dpp signal interpretation.** In cells where Brat expression is low or absent, Dpp signal is transduced by Mad-Med or Mad-Med-Shn to activate or repress transcription of Dpp target genes. When Brat expression is high, Pum-Brat complexes post-transcriptionally repress *Mad*, *Med* and *shn* mRNAs by recruiting the CCR4-NOT deadenylase complex. The CCR4 and Pop2 deadenylases in this complex act to shorten the poly(A) tail of target mRNAs leading to transcript destabilisation and degradation. Pum might also repress translation of target mRNAs through interaction with the 5′ 7 mG cap. This interaction would block binding of eIF4E to the cap and hence prevent recruitment of other initiation factors.

In this study we provide evidence that Pum-Brat repress *Mad*, *Med* and *shn* mRNAs in CBs, preventing transduction of the Dpp self-renewal signal. Mechanistically we show that lower levels of the Pop2 deadenylase are associated with weaker Pum-Brat repression in a tissue culture assay. Consistent with this, knockdown of the Pop2 or CCR4 deadenylases in the germarium gives rise to a similar phenotype of extra GSC-like cells as we have previously reported for the *brat* mutant (Harris et al., 2011). We show that these extra GSC-like cells are a result of repression of *bam* by higher pMad, Med and Shn levels in cells outwith the niche, suggesting that these deadenylases are required in CBs to initiate timely differentiation. We present evidence that the repressed *Mad* mRNA has a shorter poly(A) tail, and we detect fewer *Mad* and *shn* mRNA molecules in CBs relative to GSCs. The simplest interpretation of these data is a model whereby Pum-Brat repression of the target mRNAs involves recruitment of the CCR4-NOT complex, resulting in poly(A) tail shortening and degradation of target transcripts, reducing Dpp signal transduction (Fig. 7). Finally, we detect expanded pMad and Dpp target gene expression in *brat* mutant embryos, suggesting that this repression also occurs at other developmental stages.

Although knockdown of CCR4 and Pop2 results in extra GSC-like cells, these cells are able to differentiate eventually as observed previously in the *brat* mutant (Harris et al., 2011). Presumably these cells are pushed further from the niche with each GSC division, so receive progressively lower levels of Dpp, which acts at short range (Harris and Ashe, 2011). Therefore, even if Mad protein continues to accumulate in CCR4/Pop2 knockdown germaria it is unlikely to be activated far from the niche because of a lack of Dpp, allowing *bam* de-repression and differentiation. Other Pum-Brat-independent mechanisms promote differentiation in CBs by attenuating the Dpp signal, including Fused/Smurf-dependent proteolysis of Thickveins (Xia et al., 2010; Casanueva and Ferguson, 2004). In addition, Dpp-independent mechanisms, such as repression of the miRNA pathway (Neumüller et al., 2008) and lower levels of translation overall (Zhang et al., 2014), also promote differentiation. As these mechanisms respond to either a reduction in Dpp signalling or are downstream of Bam (Xia et al., 2012; Neumüller et al., 2008; Zhang et al., 2014), they would be inactive in the ectopic CCR4/Pop2 knockdown GSCs. Therefore, the number of ectopic GSCs we detect should act as a readout of the Dpp signal range in the germarium, which would be equivalent to around three cell diameters.

CCR4 has also been shown to be a Pum-Nos co-factor in GSCs and is essential for GSC self-renewal (Joly et al., 2013). In *CCR4* mutants GSCs are lost to differentiation over time, even though they do not upregulate *bam* expression, eventually resulting in empty ovarioles. This has been attributed to loss of translational repression of Mei-P26 and possibly other differentiation factors by Pum-Nos (Joly et al., 2013). In contrast, we observed that knockdown of CCR4 in CBs blocked Bam de-repression and differentiation which we interpret as being as a result of higher Mad, Med and Shn levels following loss of Pum-Brat repression of the target mRNAs. We suggest that as CCR4 depletion in the CB does not affect GSC maintenance, GSCs would divide as normal. However, the daughter with reduced CCR4 activity does not upregulate Bam. *CCR4 bam* double mutants give rise to a mix of the *bam* mutant phenotype of tumorous undifferentiated cells with the germline loss phenotype characteristic of the *CCR4* mutant, with the latter prevailing over time (Joly et al., 2013). Therefore, although *CCR4 bam* double mutant germline cells can differentiate, the process appears to be slower than normal, which could explain why we capture extra GSC-like cells when we knock down *CCR4* in the CB.

As discussed above, our data suggest a model whereby Pum-Brat recruit deadenylases resulting in a shorter poly(A) tail and subsequent destabilisation of target transcripts. However, we also find that complete Pum-Brat repression depends on a conserved Trp residue W783, which allows Pum to interact with the 5′ 7 mG cap and hence block translation initiation in other species (Cao et al., 2010). Therefore, we cannot rule out that the Pum-Brat repression mechanism is primarily through effects on translation initiation, via an interaction of Pum and the 5′cap which prevents eIF4E recruitment (Fig. 7), and that subsequent CCR4-NOT complex recruitment and deadenylation is potentially a secondary effect. This scenario would be similar to Pum-Brat-Nos repression of *hb* mRNA, as although there is evidence for deadenylation of *hb* mRNA (Wreden et al., 1997), this appears to be secondary to the repression mechanism (Chagnovich and Lehmann, 2001). To add to this complexity, Brat itself has recently been shown to mediate both translation repression and degradation of mRNAs, including maternal mRNAs, in the embryo (Laver et al., 2015). We note that a complex relationship also exists between translational repression and mRNA deadenylation/degradation with respect to miRNA regulation (Wilczynska and Bushell, 2015). Controversy exists over whether translation repression precedes deadenylation (Meijer et al., 2013) or deadenylation is dominant (Eichhorn et al., 2014), with the added complexity of context-dependent effects (Subtelny et al., 2014). Overall, these studies highlight the difficulties that have been encountered in defining the order and primary target of post-transcriptional control mechanisms, with multiple effects usually observed.

Another possibility for the function of CCR4 in CBs is that it is independent of deadenylation, given the precedents for this in other contexts. CCR4-NOT is recruited to miRNA regulated mRNAs via GW182 (also known as Gawky) proteins (Fabian et al., 2009; Jinek et al., 2010), and can repress poly(A)^−^ as well as poly(A)^+^ mRNAs, suggesting that CCR4-NOT can inhibit translation independently of deadenylation (Chekulaeva et al., 2011). Similarly, the Pop2 homologue CAF1 has been shown to repress poly(A)^−^ mRNAs in *Xenopus* oocytes and Pop2 lacking deadenylase activity retains some ability to repress cap-dependent translation initiation (Cooke et al., 2010). It is clear that further studies are required to precisely define the roles of CCR4 and Pop2 in regulating post-transcriptional repression in the CB.

We observed expanded expression of pMad and Dpp target genes, with associated increased numbers of amnioserosa cells, in *brat* mutant embryos, suggesting that Brat is required to limit the range of Dpp signal transduction by post-transcriptionally repressing *Mad* mRNA and, by extrapolation, *Med* and *shn* mRNAs. As Mad protein accumulates in a wild-type embryo when Brat is present, this suggests that repression is normally incomplete. This might relate to relative levels of the maternally expressed target mRNAs and that of the Brat protein and/or other members of the repression complex. Alternatively, it is possible that a feature of post-transcriptional repression during development is that it is used for fine-tuning protein levels rather than for a complete shutdown of protein synthesis. This is the case for Pum-Nos repression of *mei-P26* mRNA in GSCs, with a particular level of Mei-P26 protein associated with GSC self-renewal (Li et al., 2012), whereas high Mei-P26 promotes differentiation (Neumüller et al., 2008).

We suggest that repression of *Mad* mRNA in the embryo also involves Pum, so that the repression mechanism would be the same as we observed in S2 cells and in the ovary, which both implicate Pum (Harris et al., 2011). However, it has been suggested that the *Mad* 3′ UTR can be repressed by Brat alone in S2 cells, independently of Pum (Loedige et al., 2014), and *Med* mRNA has recently been identified as a Brat but not Pum target in the embryo based on RIP-seq analysis (Laver et al., 2015). The reason for this discrepancy is unclear but it is possible that Brat could inhibit *Mad* or *Med* mRNA translation in the absence of Pum in some circumstances, for example in the embryo where we hypothesise partial repression occurs as described above, although optimal repression might require both Pum and Brat. Consistent with this, Brat has recently been shown to function largely independently of Pum in the embryo, although some mRNAs are co-regulated by both (Laver et al., 2015). Brat has also been implicated in repression of Mad and hence modulation of BMP signalling at neuromuscular junction (NMJ) synapses (Shi et al., 2013). Given the differences in the NMJ phenotypes associated with the *pum* and *brat* mutants (Shi et al., 2013; Menon et al., 2004), the NMJ synapse might represent one context where Brat repression of Mad is Pum-independent. However, the same study also suggested co-repression of Mad by Brat and Pum in presynaptic neurons (Shi et al., 2013), identifying another situation where Pum and Brat potentially cooperate to repress Mad.

A major way in which a cell responds to changes in its environment, including chemical signals, is through the coordinated regulation of sets of genes at the transcriptional level. This varies in complexity from the co-regulation of genes arranged in operons within the genomes of prokaryotes, *C. elegans* and plants (Ishihama, 2010; Blumenthal, 2004; Boycheva et al., 2014) to the more complex coordination of whole gene expression programmes by particular transcription factors (Bonn and Furlong, 2008). At the level of translation, there is recent evidence for colocalisation of mRNAs encoding components of protein complexes or pathways within particular cytoplasmic granules that might allow coordinated translation (Lui et al., 2014; Gao et al., 2015). Therefore, a logical extension of these findings would be the coordinated post-transcriptional repression of mRNAs encoding proteins within specific complexes or pathways to limit their time of action. Our data, identifying the mRNAs encoding all three components of the Mad-Med-Shn complex as targets of Pum-Brat post-transcriptional repression, support this idea. This tripartite regulation might represent an important fail-safe mechanism to ensure that Dpp signal transduction is blocked efficiently in the CB, permitting differentiation despite proximity of this cell to the niche. In contrast, as discussed above, in the early embryo it appears that partial repression modulates interpretation of the Dpp signal. In this way, post-transcriptional repression of the Dpp signal transducers can act as a ‘volume’ control, offering great flexibility in regulating the strength of the signal response either across a field of cells or more specifically within individual cells depending on the levels of Pum-Brat. We predict that post-transcriptional regulation of specific components of other signalling pathways will be widely used as a mechanism to refine the cellular response during development.

## MATERIALS AND METHODS

### Fly stocks

Fly stocks used were: *yw^67c23^* (used as wild type in all experiments); *bam-GFP* (Chen and McKearin, 2003b); *Pop2*, *P{TRIP.HMJ21614}* and *twin*, *P{TRIP.HMS00493}* shRNA (Harvard Medical School TRiP project, Bloomington Stock Center); *bam-Gal4* (Chen and McKearin, 2003b); *Med^14^/TM3*, *PBac{Med-GFP.FLAG}* and *PBac{Shn-GFP.FLAG}* (Bloomington Stock Center). *brat^11^* clonal germ lines were generated by FLP/FRT recombination as described previously (Harris et al., 2011). *brat^11^* germline clone embryos were generated using the FLP-DFS system: larvae of genotype *hs-FLP*; *brat^11^-FRT*/*Povo^D^-FRT* were heat shocked for 3 h on two consecutive days, adult females were then crossed to *brat^11^-FRT*/*CyO* males and the resulting embryos were collected.

### *In situ* hybridisation and immunofluorescence

Embryos were collected from yeast/apple juice plates and fixed and stained using standard techniques. Ovaries were dissected from adults after maturing on yeast/apple juice plates and fixed according to standard techniques. Primary antibodies used were: anti-pSmad3 (for pMad, 1:1000, Epitomics 1880-1); rabbit anti-GFP (1:200, Torrey Pines Biolabs TP401); mouse anti-spectrin [1:20, DSHB Hybridoma Product 3A9 (323 or M10-2), deposited to the DSHB by D. Branton, R. Dubreuil]; mouse anti-Bam (1:20, DSHB Hybridoma Product bam, deposited to the DSHB by D. McKearin); mouse anti-Hnt (1:20, DSHB Hybridoma Product 1G9, deposited to the DSHB by H. D. Lipshitz); rabbit anti-Bam (1:200, a gift from D. Chen, Chinese Academy of Sciences, China); rabbit anti-Med (1:1000, Sutherland et al., 2003) and rabbit anti-Mad (1:3000, a gift from Julia Zeitlinger, Stowers Institute, USA). Secondary antibodies used were donkey anti-mouse and donkey anti-rabbit Alexa488 (Life Technologies A21202) and Alexa594 (Life Technologies A21207) and goat anti-mouse-AP (Promega S372B) (all used 1:500). Stellaris probes (Biosearch Technologies) were designed to detect *Mad*, *shn* and *Tbp* mRNAs using the Biosearch design tool. 48 probes were designed and labelled with Quasar^570^ dye. Probes were hybridised at 37°C overnight and ovaries were co-stained with anti-GFP during this incubation. Images for Stellaris FISH were obtained using a Delta Vision microscope and deconvolved using Softworx software. Images were then quantified using Imaris software; the ‘Surfaces’ function was used to create a 3D mask around each GSC or CB and the number of Mad transcripts within each masked region was quantified using the ‘Spots' function in the red channel.

### Tissue culture and western blot

S2 cells were cultured in Schneider's Modified Drosophila Media (Invitrogen) supplemented with 10% FBS and 1% penicillin/streptomycin. DNA constructs for transfection were generated using standard techniques and the PumW783G mutation and mutations in Pum and Brat binding sites in *Mad*, *Med* and *shn* 3′ UTRs were generated by site directed mutagenesis using QuikChange (Stratagene). In each case the first four nucleotides of the Pum sites were mutated to ACGC and all U nucleotides in the Brat sites were mutated to A. Double-stranded RNAs were synthesised using the T7 MEGAscript kit (Ambion) according to the manufacturer's instructions. For RNAi knockdown cells were incubated with 5 µg dsRNA for 24 h prior to transfection. DNA constructs were transfected using Effectene (Qiagen) according to the manufacturer's instructions and cells were harvested following three days' expression. Luciferase assays were carried out using the Stop+Glo Dual Luciferase kit (Promega) according to the manufacturer's instructions. Western blots were performed using standard methods and detected using Li-Cor Infrared detection system. Primary antibodies were used at 1:2000 dilution: rabbit anti-GFP (Abcam Ab290), mouse anti-tubulin (Abcam Ab44928) and mouse anti-V5 (Abcam Ab27671) and 1:1000 dilution: rabbit anti-CCR4 and rabbit anti-Pop2 (Temme et al., 2010). Secondary antibodies were used at 1:10,000 dilution: IRDye 800CW donkey anti-mouse (Li-Cor 926-32212) and IRDye 680RD donkey anti-rabbit (Li-Cor 926-68073).

### qPCR and RACE-PAT assay

S2 cells were transfected and harvested as described above. RNA was extracted using TRIZOL according to the manufacturers' instructions. Samples were treated with DNaseI (Ambion) and reverse transcribed using Enhanced Avian Reverse Transcriptase (Sigma) according to the manufacturers' protocol. qPCR was carried out by standard curve method using SYBR Green with primers for GFP and RP49 as a control. RACE-PAT assay PCR was carried out as described in Sallés and Strickland, 1999 and resulting products were run on 1% agarose gels. Briefly, reverse transcription of total cellular RNA with a 5′-anchored oligo(dT) primer resulted in a heterogeneous pool of cDNAs primed at all possible positions along the poly(A) tail. Subsequent PCR amplification with the 5′-anchored oligo(dT) and a message specific primer generated a range of different sized products representing the length of the test mRNA poly(A) tail. Specific primers used were: Mad (5′-GCAAACAAATCGAAAACATCA); betaTub (5′-GCTGAGGTCGACGAGAACTAA).

### Statistical analysis

Western blot IR signals were quantified using Li-Cor Odyssey software and qPCR signals were quantified using Opticon Monitor3 software. Error bars represent the standard deviation (s.d.) of at least three biological repeats with Student's *t*-tests used for significance.
